# The Realization of a Three-Dimensional Temperature Measurement System with a Two-Dimensional Sensor Array and the Demonstration of the Deformation Effect of Gravity on the Heating Patterns

**DOI:** 10.3390/s25010198

**Published:** 2025-01-01

**Authors:** Dogan Can Samuk, Oguzhan Cakir

**Affiliations:** 1 Department of Electrical and Electronics Engineering, Faculty of Engineering and Architecture, Recep Tayyip Erdogan University, Rize 53100, Türkiye; dogancan.samuk@erdogan.edu.tr; 2 Department of Electrical and Electronics Engineering, Faculty of Engineering, Karadeniz Technical University, Trabzon 61080, Türkiye

**Keywords:** gravity deformation effect, temperature measurement system, heating patterns, three-dimensional heating patterns

## Abstract

Electric heaters are widely used owing to their portability, fast heating, single-focus heating, and energy efficiency advantages. Manufacturers provide customers with information on the power consumption and energy efficiency classes of heaters but do not provide any information on heating patterns. Knowing the heating pattern enables users to select the correct heater, which has a significant effect on comfort, health, energy efficiency, industrial process performance, plant growth, and climate change. In previous studies, two-dimensional temperature measurements were performed using sensor arrays. However, the three-dimensional heating patterns of the heaters have not been extracted, and the deformation effect of gravity on the heating patterns has not been demonstrated. In this study, a temperature measurement system with 64 temperature sensors placed at equal intervals in the *xz*-plane was designed and implemented. Then, the fan heater was moved along the *y*-axis at intervals of 10 cm from 0 to 100 cm, and three-dimensional heating patterns were obtained for different fan voltages. As part of the research objectives, the deformation effect of gravity on the heating pattern was revealed, and the shift in the maximum temperature point on the +*z*-axis was measured. The mathematical formula for the maximum temperature value was derived based on the fan voltage and the distance between the heater and the sensor array. The goodness-of-fit statistical values for the derived mathematical formula for the 55 temperature measurements were calculated as the root mean square error of 1.9543 and R-squared of 99.43%, demonstrating the accuracy of the presented model.

## 1. Introduction

Electric heaters have a wide range of applications in both industry and daily life. They are used in homes and offices when central heating systems are inadequate or extra heating is required. Electric heaters are preferred for local heating in factories, warehouses, workshops, and industrial areas or to provide additional heating in special processes. In addition, outdoor seating areas utilize heaters to increase customer satisfaction. Electric heaters are also used in greenhouse cultivation to protect plants and crops from cold temperatures.

Different types of electric heaters are produced depending on their intended use, energy efficiency, and the characteristics of the environment to be heated. Radiant heaters are used for quick and focused heating, whereas fan heaters are preferred for homogeneous heating. Convection and ceramic heaters are used in areas where comfort is prioritized owing to their quiet operation. In terms of energy efficiency, halogen heaters provide rapid heating, whereas oil-filled radiators provide prolonged heating.

When selecting a heater based on its usage and purpose, factors such as the heating capacity, energy efficiency, safety features, ease of use, price and quality balance, noise level, warranty and service, heater type, and portability should be considered. Additionally, the heating patterns of the heaters, which refers to how the heat produced by a heater is distributed in the environment and how different areas are heated, are of critical importance. This depends on the design, technology, and environmental conditions of the heater. However, the absence of this information from manufacturers leads customers to incorrect heater selection. Knowing the heating pattern helps to predict which areas of the space will be warmer or cooler. This enables heating systems to operate more effectively, thereby increasing comfort and energy efficiency. Furthermore, it allows for cost-effective and high-performance heating by preventing unnecessary heating and cooling. Given the importance of optimizing the performance of heating systems, it is necessary to know the heating patterns of heaters to evaluate their performance.

In the literature, studies on temperature measurement with multiple sensors exist; however, the three-dimensional heating patterns of heaters have not been extracted, and the effect of gravity on heating patterns has not been investigated. Gravity adversely affects heating patterns by causing warm air to rise and forming cold areas in the lower parts of the environment to be heated. This results in a temperature imbalance, reduced comfort, and increased heat loss, which lowers the energy efficiency [1]. In one of the previous studies with multiple sensors, Jain and Grimes utilized four wireless, passive, magnetoelectric sensor arrays adhered to a printed circuit board for simultaneous measurement of ambient pressure and temperature [2]. With the aim of indicating the effectivity of a low-cost thermal imager, Schaufelbühl et al. presented a 10 × 10 array of thermoelectric infrared sensors with 530 mK accuracy integrated on a silicon circuit board with an area of 5.5 mm × 6 mm [3]. In another study using a multiple sensor array, Nguyen measured the wind speed and direction with 40 thermocouples in an 8 × 5 array placed on a 6 cm × 6 cm surface according to the polar coordinate system [4]. In a study with a two-dimensional temperature profile, Nguyen offered a novel thermal flow sensor [5]. He measured the ambient temperature with 10 temperature sensors placed at equal intervals in a 1 m long fiber optic cable. Ivanov et al. performed temperature measurements up to 400 °C with temperature sensors placed at equal intervals in a 15 mm long fiber optic cable through a differential coherent multiplexing technique [6]. Using 20 temperature sensors positioned in a fiber-optic cable, Tao presented a measurement system to detect leakage in a dam body [7]. With the aim of accurate thermal reading, Luria and Shor observed hotspots on a microprocessor with 20 nanometric temperature sensors [8]. In this direction, they ended up with a compact thermal sensor for better performance in processors. A survey conducted by Pfrimer achieved high-accuracy temperature measurements in a laboratory environment using two temperature sensors placed in a fiber cable [9]. Ge et al. proposed a fire localization system with two sensor arrays, each consisting of four wireless temperature sensors [10]. In a different context, Zacepins and Meitalovs implemented a measurement system consisting of 10 temperature sensor nodes for online monitoring of bee colonies [11]. Regarding the environmental domain, Oiler et al. designed and implemented a measurement system with 15 sensors to observe the parameters of the hot springs [12]. Zbiec and Obrebski have presented the design and testing process of a 12-sensor system for temperature measurement in an aerodynamic tunnel [13]. Hariharan et al. have determined the position of a focused ultrasonic beam in tissue using 3, 4, or 5 thermocouples [14]. In one of the previous research studies, Bouderbala et al. measured the temperature of a mechanical guidance system using 19 PT100 sensors [15]. Using a fiber optic cable containing 213 temperature sensors, Bazzo et al. measured the heat distribution in the stator of a 200 MW hydroelectric generator [16]. Billard et al. designed and tested a neural probe with eight temperature sensors for chronic measurements and recording of localized temperature areas in the brain [17]. In a similar domain, Chi measured four different psychological cell parameters and drug delivery using 16 temperature sensors placed on a 2.2 mm × 2 mm silicon circuit board [18]. Reigosa et al. estimated the temperature distribution of a continuous magnet synchronous machine with 90 thermocouples embedded in its rotor [19]. Morona et al. measured the temperature of a reactor online using a fiber-optic cable with five temperature sensors inside [20]. In the case of posture detection, Russell et al. tracked the appropriate seating position through 30 temperature sensors placed on a chair [21]. Linder used four temperature sensors to observe the temperature distribution during aluminum casting within the cooling and solidification processes [22]. In a proof-of-concept study, Cheng designed a high-speed and high-resolution demodulation system for a fiber microstructure sensor network with 16 temperature sensors [23]. Presenting the inefficacy of the current thermal response tests for depth-dependent temperature measurements, Aranzabal et al. proposed a measurement system with seven temperature sensors to observe underground thermal properties to increase the energy efficiency and economic feasibility of ground-source heat pump systems [24]. Responding to the need for a PV array operating at different spatial conditions, Escribano et al. used a nine-sensor measurement system to determine spatial temperature differences in photovoltaic panels [25]. A recent work by Zhang et al. proposed a measurement method using eight temperature sensors to detect liquid nitrogen levels under dynamic conditions [26]. Recently, investigators have examined the effects of designing a wireless ambient-temperature measurement system on accurate meteorological measurements. In the study, Panpan et al. presented a fusion system using three temperature sensors [27]. Previous research conducted by Geczy et al. measured the vapor and heat distributions of a vapor-phase soldering station using nine K-type thermocouples placed in a two-dimensional plane [28]. They have contributed to the literature by offering a calibration procedure for the identification of vapor and heat propagation. Results from an earlier study by Jakovenko et al. indicated that simulation models could be used to estimate measured temperatures [29]. They modeled an 8 W LED lamp in ANSYS-CFX and ConvertorWare programs and computationally simulated its heat dissipation in the environment. In addition, they measured the heat emitted by the lamp using four thermocouples. In one of the recent studies in this field, Liu et al. proposed a new method for measuring two-dimensional flame temperature distribution based on element doping and energy spectrum analysis [30]. Taking into consideration the importance of accurate temperature measurement in the medical sector, Gaspar et al. implemented a system to measure skin temperature using digital temperature sensors [31]. Vincent et al. introduced a smart cell monitoring system using 21,700 internal cells equipped with a built-in array of seven thermistors [32]. Their circuit system promised an excellent message error rate and no need for additional wiring for data transmission. A comprehensive review of the aforementioned studies revealed that temperature measurements using multi-sensor arrays have diverse applications across various fields. These applications include environmental monitoring, the industrial and medical sectors, renewable energy, and power systems. These studies were conducted using multi-sensor arrays [1,2,3,18,28,29], single-row arrays [4,5,6,19,22], and small-scale sensor arrays [7,16,17,28] and have been performed for purposes such as temperature measurement, thermal imaging, temperature profiling, and biological temperature monitoring. Sensor arrays have also been used for environmental [11,23,26] and structural monitoring [12,15,20,21]. For industrial and energy applications, sensor arrays have been utilized for various purposes, such as temperature distribution [24,25] and monitoring [14,27]. The literature also indicates that temperature sensors have been employed for medical [30] and biological applications [10]. Despite numerous studies conducted to date, none have focused on extracting the three-dimensional heating patterns of heaters using a two-dimensional sensor array. The production of a three-dimensional measurement system involves many challenges owing to hardware complexity, data management, and software development requirements. The integration of sensors complicates field and energy management, whereas issues such as large data volumes and signal interference make data processing more complex. All these factors have made the design and production of three-dimensional measurement systems that enable the extraction of heating patterns a complex process, which is why no studies have been conducted on the extraction of heating patterns to date.

In this study, a measurement system consisting of 64 sensors was implemented to extract the three-dimensional heating patterns of the electric heaters. The sensors were arranged in a two-dimensional 8 × 8 array within the measurement system, and the heating patterns were obtained through the horizontal movement of the heater across the sensor array. A fan heater was used in the experimental setup, and graphs illustrating the disruptive effect of gravity on the heating patterns were presented. To be specific, the novel contributions of this study are summarized as follows:

- The three-dimensional heating pattern of the fan heater was extracted using 64 temperature sensors arranged in an 8 × 8 layout in the *xz*-plane;
- The deformation effect of gravity on the three-dimensional heating pattern was demonstrated;
- A three-dimensional model of the maximum temperature point was developed based on the fan voltage and distance between the heater and the sensor. The obtained model was compared with the measurement results to demonstrate its accuracy;
- The effect of gravity on the maximum temperature point shifting towards the +*z* axis was analyzed based on the fan speed and heater–sensor distance.

The remainder of this paper is organized as follows. In Section 2, a three-dimensional temperature measurement system is introduced. Section 3 describes the experimental setup in detail. The experimental results are discussed in Section 4, and the proposed mathematical model is verified. Finally, Section 5 concludes this study.

## 2. Materials and Methods

Electronic and fiberoptic measurement systems are used in applications involving multi-sensor temperature measurement [33]. Fiberoptic temperature measurement systems offer significant advantages such as immunity to electromagnetic interference, high precision, and suitability for harsh environmental conditions [34,35]. However, the implementation of these systems requires precise components and technical expertise. However, electronic temperature measurement systems are advantageous in terms of the diversity of their components, ease of access, seamless integration with existing systems, and cost-effectiveness [36]. Therefore, electronic components are often preferred in measurement systems using 2D sensor arrays. However, electronic measurement systems may exhibit measurements owing to signal processing and data transmission times. In addition, unlike fiberoptic systems, systems integrating electronic sensors are more sensitive to electromagnetic interference.

In the temperature measurement system, the PT100 sensor, classified as an electronic sensor, was preferred owing to its high accuracy, stability, reliability, and fast response time. The temperature sensor has a validity class of F 0.3 and is manufactured by JUMO [37]. To mitigate the disadvantages of electronic temperature measurement systems, 64 temperature sensors were measured simultaneously and in parallel, using four control units. In addition, shielded signal cables were used to enhance the noise immunity of the system. Furthermore, the acquired analog temperature signals were filtered to suppress the noise. This section introduces the proposed temperature measurement circuit and the implemented temperature measurement system.

### 2.1. Temperature Measurement Circuit

The temperature measurement circuit consists of a voltage divider resistor in series with a PT100 temperature sensor. In this circuit, *V_CC_* is the supply voltage, *V_S_* is the sensor voltage, *R*_1_ is the voltage divider resistor, and *R_S_* is the resistance of the PT100 temperature sensor [38,39,40,41,42]. In the design of the circuit, when determining resistance *R*_1_, factors such as the maximum sensor current, reference voltage (*V_R_*), and measurement accuracy should be considered. The maximum sensor current was 7 mA for the PT100 temperature sensor used in this study [43]. *V_R_* is the reference input voltage of the internal analog-to-digital converter (ADC). The smaller the *V_R_*, the higher the resolution of the measurement. However, if the *V_R_* is too small, the system will become more susceptible to noise in the environment. Ideally, the *V_R_* should be equal to the maximum sensor voltage. *V_R_* can be adjusted using an external voltage source or can be selected using the embedded software of the microcontroller.

The *V_S_* sensor voltage can be calculated using (1).

(1) $$V_{S}=V_{CC}\frac{R_{S}}{{R_{1}+R}_{S}}\left[V\right],$$

Because *R_S_* varies directly proportional to the temperature, the sensor resistance corresponding to the lowest temperature value in the measurement range must be considered when calculating the maximum sensor current (*I_Smax_*). The measurement range of the implemented three-dimensional temperature measurement system ranged from 0 °C to 250 °C. In this case, the maximum sensor current is calculated using (2).

(2) $$I_{Smax}=\frac{V_{CC}}{R_{1}+R_{0}}\;\;\left[A\right],$$

where *R*_0_ is the resistance of the PT100 temperature sensor at 0 °C.

The resolution has a significant impact on the measurement accuracy. The smaller the resolution of a measurement system, the more precise and accurate the measurements that can be made. Depending on the resolution of the ADC, the voltage per bit can be calculated using (3).

(3) $$V_{bit}=\frac{V_{R}}{2^{n}}\;\;\left[V\right],$$

where *n* represents the resolution of the ADC. The resolution of the measurement system (*Res*) can be determined using (4).

(4) $$Res=\frac{250\;{}^{\circ}C\;}{V_{250}-{\;V}_{0}}V_{bit}\;\;\left[{}^{\circ}C\right],$$

The resistances of the PT100 sensor at 0 °C and 250 °C are 100 Ω and 194.1 Ω, respectively [44]. The *V_CC_* = 5 V, *R*_1_ = 1500 Ω, and *V_R_* = 0.6 V were selected in the sensor circuit. *V_S_* was sampled using a 10-bit ADC. In this case, according to (2)–(4), *I_Smax_* = 3.125 mA, and the resolution was calculated as 0.56 °C.

### 2.2. Temperature Measurement System

**Figure 1:**
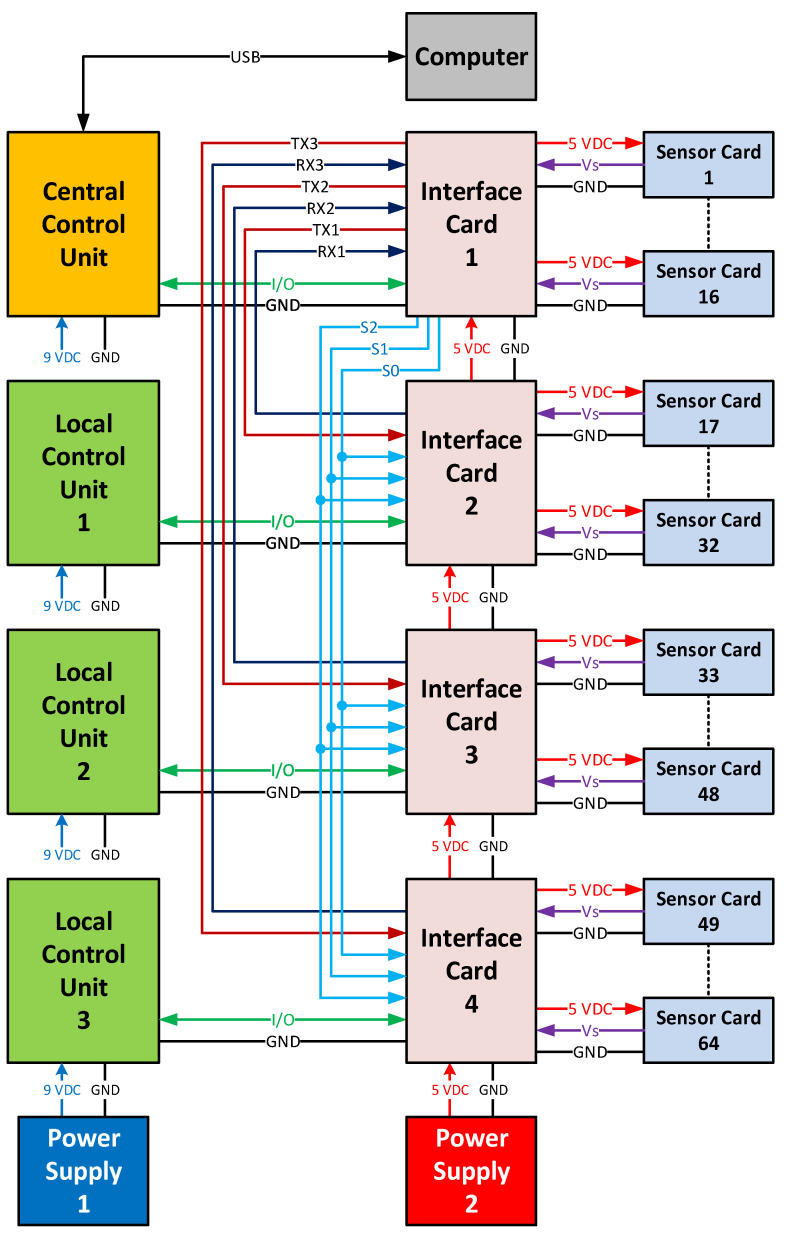
Block diagram of the temperature measurement system.

A block diagram of the temperature measurement system is shown in Figure 1. The measurement system comprised one central control unit (CCU) and three local control units (LCUs).

The CCU accesses all LCUs via UART ports (UART1–UART3) and transfers the measurement data to the computer via USB. In this data transfer, the CCU operates in master mode, while the LCUs operate in slave mode. The CCU simultaneously initiates the sampling process for all LCUs. At the end of sampling, it sequentially retrieves the measurement data from the LCUs and transfers them to the computer. The input–output pins and commands for sampling and communication control are provided in Table 1.

**Figure 2:**
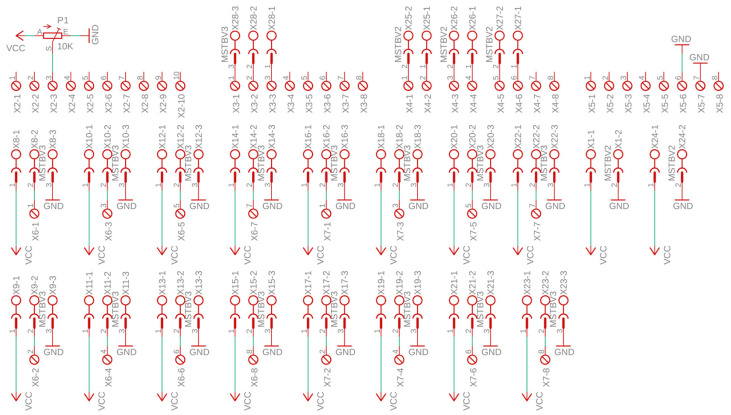
Schematic diagram of the interface card.

**Figure 3:**
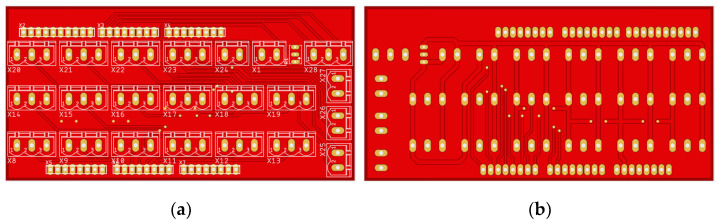
Printed circuit board of the interface card: (**a**) top; (**b**) bottom.

The connection between the sensor cards and controllers was provided by the interface cards. The measurement system obtained the two-dimensional temperature pattern of the heater using 64 sensors. There were four identical interface cards in the system, each connected to 16 sensor cards. A schematic of the interface card is provided in Figure 2, and a printed circuit diagram is shown in Figure 3. In the schematic, the X8–X23 connectors indicate the connection points of the sensor cards. The analog outputs of the 16 sensor cards were connected to the 16 analog inputs of the controller through X6 and X7 connectors.

**Figure 4:**
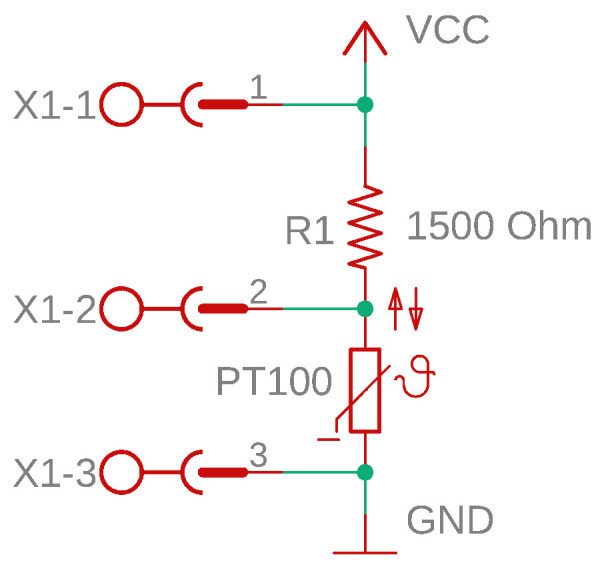
Schematic diagram of the sensor card.

Interface cards facilitated the transmission of communication signals between the controller cards. Each controller had four built-in UARTs. In the measurement system, serial communication pins TX0 and RX0 of UART 0 were used to communicate with the computer via the USB port. In the schematic shown in Figure 4, the communication pins of the 1st, 2nd, and 3rd UARTs were connected to the X4 connector as TX3, RX3, TX2, RX2, TX1, and RX1, respectively. Communication control between the central controller and local controllers was facilitated by three digital input–output terminals connected to the X3 connector, as depicted in Figure 2.

The VR was generated with the 10 kΩ multi-turn P1 potentiometer, as shown in the schematic diagram in Figure 2, and connected to the ADC via the X2 connector. The 5V supply voltage was distributed to the controllers through the interface cards. The supply voltage entered the interface card from X1 connector, as shown in the schematic diagram in Figure 2, and was transmitted to the other interface card through X24 connector.

**Figure 5:**
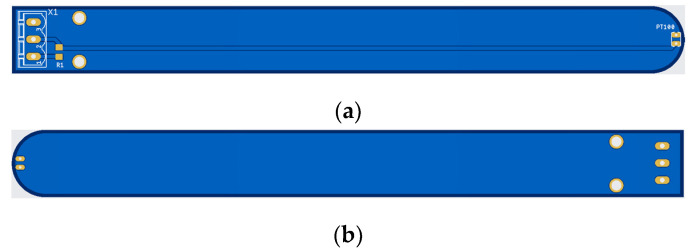
Printed circuit board of the sensor card: (**a**) top; (**b**) bottom.

A schematic of the sensor card prepared in the Autodesk Eagle editor is shown in Figure 4, and the printed circuit layout is shown in Figure 5 [45]. The connection between the sensor and interface cards was provided through the X1 connector, as shown in Figure 4. Pin 1 of the X1 connector was the voltage output of the second power supply. The second pin of the connector was the sensor voltage output. The third pin was the DC ground terminal of the second power supply.

## 3. Experimental Setup

**Figure 6:**
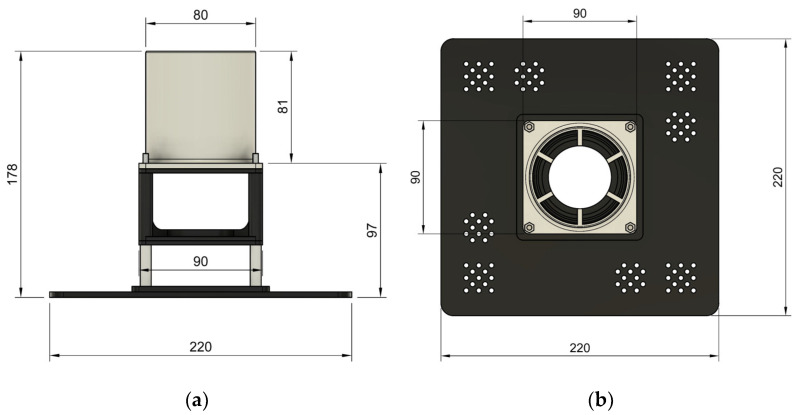
Dimensions of the fan heater in mm: (**a**) right view of the 3D model of the fan heater; (**b**) top view of the 3D model of the fan heater.

The fan heater shown in Figure 6 was designed using Autodesk Fusion 360 (Autodesk, Inc., San Francisco, CA, USA) and printed on a three-dimensional resin printer. Inside the fan heater, there was a fan motor operating between 12 and 24 V and a resistor consuming 900 W of power at a grid voltage (220 VAC).

**Figure 7:**
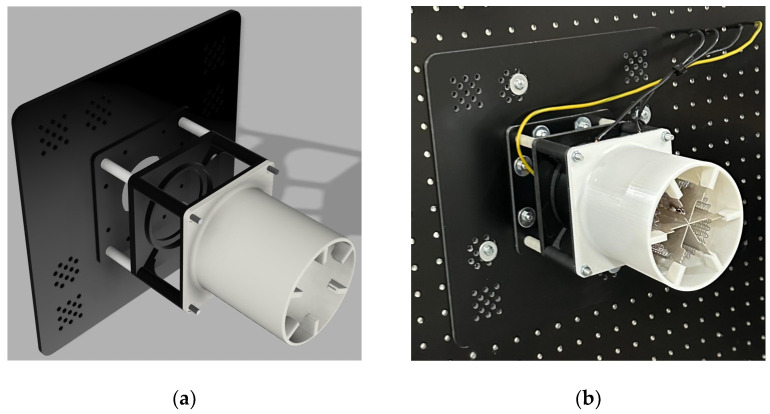
Fan heater: (**a**) perspective view of the 3D model of the fan heater; (**b**) perspective view of the fan heater.

The fan heater was mounted on the measurement table using a mounting adapter with dimensions of 220 mm × 220 mm. The three-dimensional design and perspective view of the heater are shown in Figure 7.

**Figure 8:**
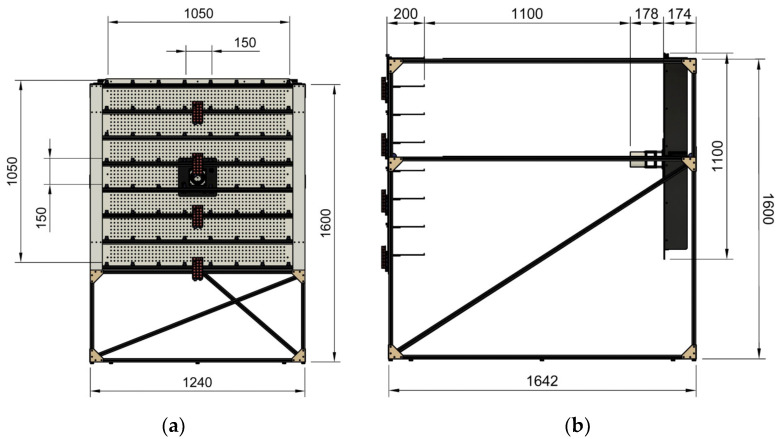
Dimensions of the measurement system in mm: (**a**) back view of the 3D model of the measurement system; (**b**) right view of the 3D model of the measurement system.

The measurement table on which the heater was placed is shown in Figure 8a. The table contained 2601 mounting holes arranged in 51 rows and 51 columns at intervals of 20 mm. The heater was mounted on the holes using a mounting adapter. At each corner of this adapter, there were 25 mounting holes, ensuring precise placement of the heater on the table with a tolerance of 5 mm. Mounting holes with a diameter of 4 mm were drilled into two groups, one with 13 holes and the other one with 12 holes, to prevent them from overlapping.

There was 1100 mm between the fan heater and the sensors, as shown in Figure 8b. The 64 sensors placed in the *xz*-plane were fixed, and the table was moved along the y-axis to obtain the three-dimensional (*xyz*) heating pattern of the heater.

**Figure 9:**
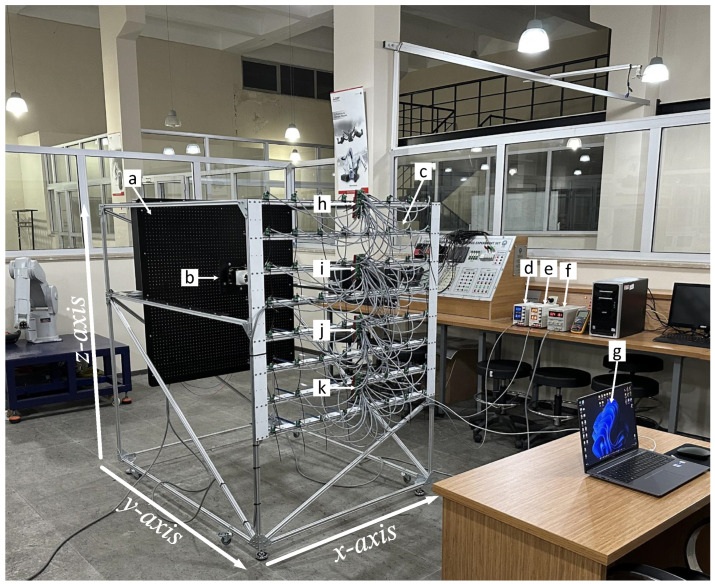
General view of the temperature measurement system: (**a**) measurement plate; (**b**) fan heater; (**c**) 8 × 8 sensor grid; (**d**) power supply 1; (**e**) power supply 2; (**f**) power supply 3; (**g**) computer (**h**) LCU1; (**i**) CCU; (**j**) LCU2; (**k**) LCU3.

**Figure 10:**
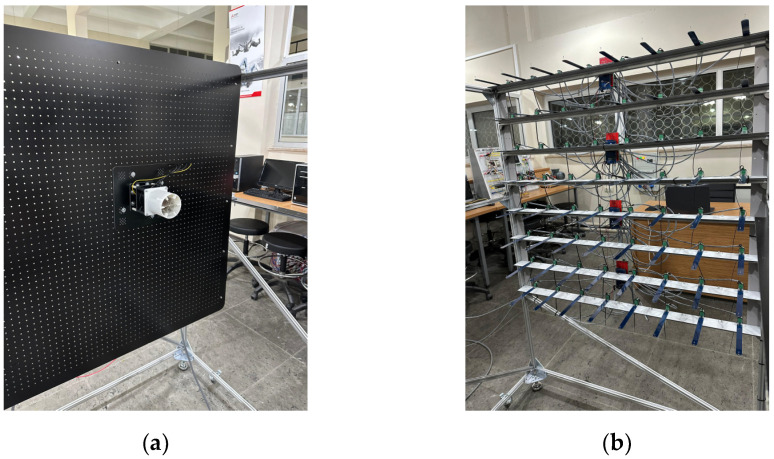
Main components of the temperature measurement system: (**a**) measurement plate; (**b**) 8 × 8 sensor grid.

Alternatively, the heater can be kept fixed, and the sensor array can be moved. However, since the two-dimensional sensor array consists of 64 sensors, there are 64 cable connections (power and signal lines) between the sensors and interface boards. In addition, power and communication cables exist between the control units. Moving the sensor array, which has a complex and delicate structure, may lead to contact issues in the cable connections and changes in the sensor positions. Furthermore, a more complex and costly movement mechanism is required to move the sensor array, which has significantly more weight and volume than the heater. Therefore, to achieve more consistent and reliable measurement results, the heater was moved towards the sensor array. The measurement system is shown in Figure 9, and its components are shown in Figure 10.

### Temperature Measurement Steps

The fan heater was placed at the center of the measuring table, and temperature measurements were performed. The measurement steps were as follows:

Step 1. The heater–sensor array distance (*d_hs_*) (0–100 cm) was set.
Step 2. A fan voltage (16–20 V) was applied.
Step 3. The resistance of the fan heater was energized with a grid voltage (220 VAC).
Step 4. The fan heater was heated for 30 s.
Step 5. Each of the 64 sensors was sampled for 20 s at a sampling frequency of 100 Hz with a 10-bit resolution, and the measurement results were stored.
Step 6. The 2000 temperature measurements for each sensor were averaged and stored.

## 4. Results and Discussions

In this section, the experimental results are discussed in three parts. First, three-dimensional heating patterns were obtained. Additionally, the deformation effects of gravity and fan voltage on the heating patterns were examined. Second, the maximum temperature value was formulated based on the fan voltage and heater–sensor array distance, and goodness-of-fit statistical values were calculated. Finally, the shift in the maximum temperature point owing to the effect of gravity was analyzed.

### 4.1. Three-Dimensional Heating Patterns

The fan heater was moved from 0 to 100 cm in 10 cm increments along the sensor array for five different fan voltages, and 55 temperature measurements were taken. The heating patterns of 30 of these measurements are presented in Table 2. In Table 2, *d_hs_* represents the distance between the fan heater and the 2D sensor array. This table presents the heating patterns obtained for the fan motor at different operating voltages and *d_hs_* values.

Three-dimensional heating patterns were obtained in MATLAB using the measurement results shown in Table 2. The distance between the sensors in the 2D array was 15 cm. The resolution of the heating patterns was increased using the MAKIMA interpolation method [46,47].

As the distance between the heater and sensor array increased, the three-dimensional heating patterns spread across the *xz*-plane, as shown in Table 2. Additionally, the heating pattern expanded in the +*z* direction owing to the ascending heated air. Moreover, the maximum temperature point shifted towards the +*z*-axis, and the symmetry of the heating patterns was disrupted. This disruption is a major issue, especially in applications where homogeneous heating is required or in processes that require focused heating. The proposed measurement system can minimize these issues by enabling the design of fan heaters to reduce the negative effects of gravity on heating patterns.

### 4.2. Maximum Temperature Curves

**Figure 11:**
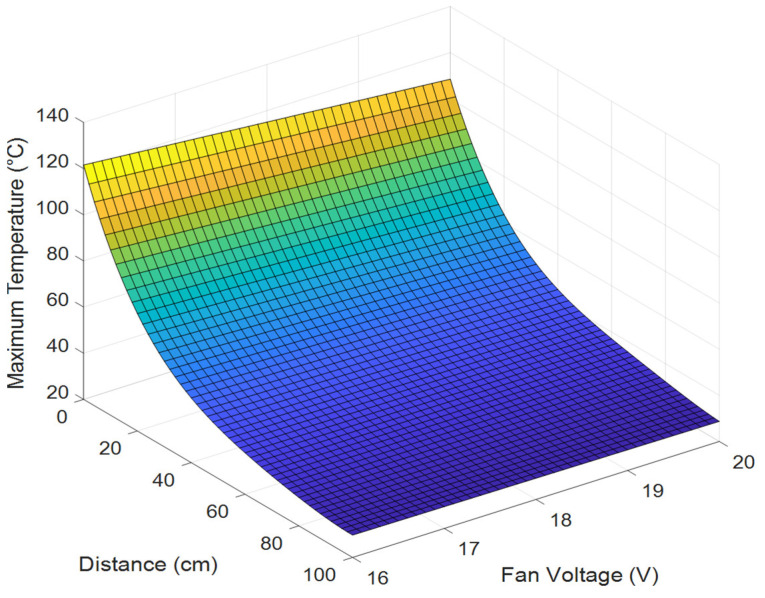
The 3D maximum temperature model.

The maximum temperature points for 30 of the 55 measurements listed in Table 2 are shown in Figure 11. As the heater–sensor distance increased, the maximum temperature exponentially decreased. Additionally, as the fan voltage increased, the airflow velocity inside the 81 mm long and 80 mm diameter guide, as seen in Figure 6, also increased. Owing to the increased velocity, the air drawn from the environment left the duct in a shorter time, causing the maximum temperature to decrease. The measured maximum temperature values were consistent with each other in terms of both the heat sensor distance and fan voltage. The maximum temperature value was obtained, as shown in (5), depending on the heater–sensor distance (*d*) and fan voltage (*v*) using the “Curve Fitter” application in MATLAB. The equation coefficients are presented in Table 3, and the goodness-of-fit statistics, root mean square error (RMSE) 1.9543, and R-squared (RSQ) 0.9943 were calculated.

**Figure 12:**
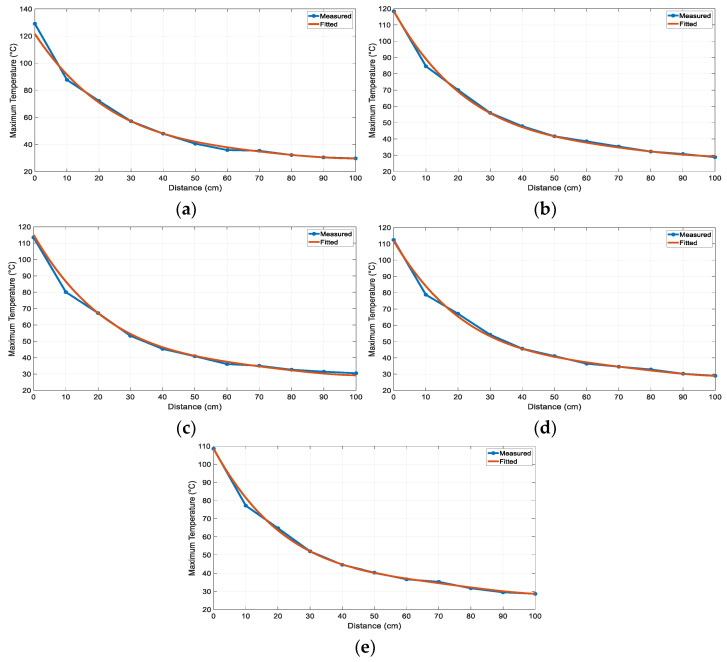
Measured and fitted curves for maximum temperature: (**a**) fan supply voltage 16 V; (**b**) 17 V; (**c**) 18 V; (**d**) 19 V; (**e**) 20 V.

The measured and calculated maximum temperature curves for each fan voltage are shown in Figure 12 to demonstrate the accuracy of (5). Additionally, the RMSE and RSQ values were calculated using (6)–(9), which are presented in Table 4.

(5) $$T_{m}=c_{1}+c_{2}d+c_{3}v+c_{4}d^{2}+c_{5}dv+c_{6}d^{3}+c_{7}d^{2}v+c_{8}d^{4}+c_{9}d^{3}v,$$

To determine the RMSE value, first, the sum of squared errors (SSE) was calculated using (6). Then, the square root of the average SSE was calculated using (7).

(6) $$SSE=\sum_{i}^{n}{\left(T_{mi}-{\hat{T}}_{mi}\right)}^{2},$$

(7) $$RMSE=\sqrt{SSE/n},$$

where *n* is the number of measurements (*n* = 11), *i* is the measurement index, *T_m_* is the measured maximum temperature, and ${\hat{T}}_{m}$ is the calculated maximum temperature. After calculating the variance of the maximum temperature ($\sigma_{T}$) using (8), RSQ was determined using (9).

(8) $$\sigma_{T}=\sum_{i}^{n}{\left(T_{mi}-{\bar{T}}_{mi}\right)}^{2},$$

(9) $$RSQ=1-\left(SSE/\sigma_{T}\right),$$

where ${\bar{T}}_{m}$ is the average of the maximum temperature measurements.

### 4.3. The Shift of the Maximum Temperature Point

**Figure 13:**
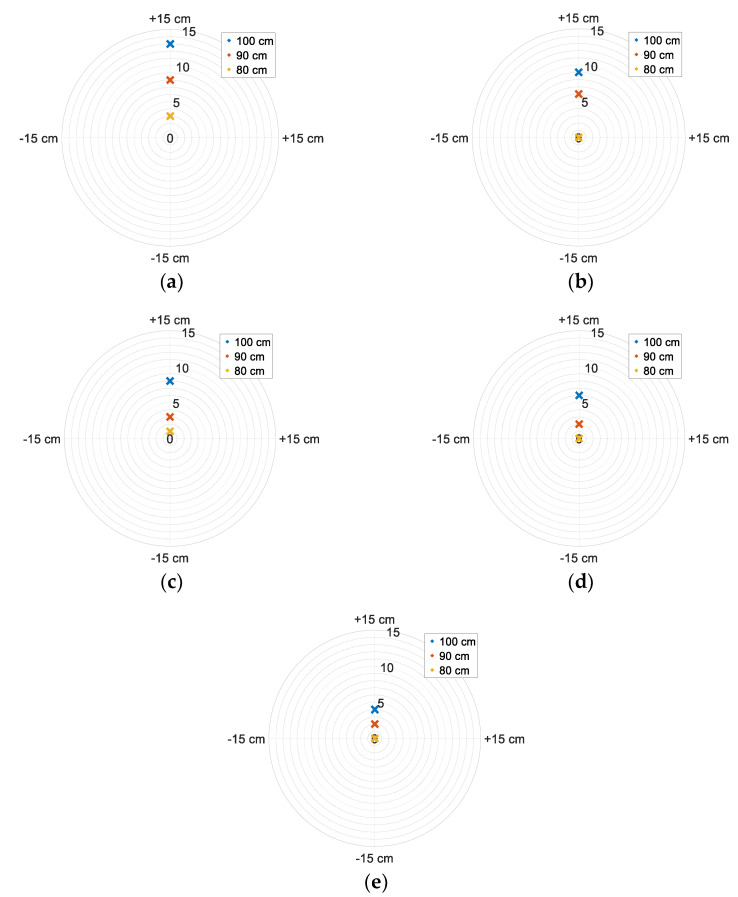
The shift of the maximum temperature point on the y-axis for heater–sensor distances of 80 cm, 90 cm, and 100 cm: (**a**) fan supply voltage 16 V; (**b**) 17 V; (**c**) 18 V; (**d**) 19 V; (**e**) 20 V.

The air passing through the fan heater expands, resulting in a decrease in its specific gravity. Therefore, it is less affected by gravity and rises. The displacement of the maximum temperature point for each fan voltage was observed for heater sensor array distances of 80, 90, and 100 cm, as shown in Figure 13. As the fan speed decreased, the time it took for the heated air to reach the sensors and the displacement of the maximum temperature point along the +*z* axis increased.

## 5. Conclusions

This study introduces a new measurement system with 64 sensors to obtain three-dimensional heating patterns of heaters. With the proposed measurement system, the three-dimensional heating patterns of a fan heater were extracted based on the distance between the heater and the sensor array. By increasing the fan voltage, the spreading of the heating patterns was reduced, providing focused heating. In addition, the deformation effect of gravity on the three-dimensional heating pattern was demonstrated. Furthermore, it was observed that as the distance between the heater and the sensor array increased, the symmetry of the three-dimensional heating pattern was disrupted.

In the process of obtaining the optimum heating pattern, a formula for the maximum temperature value was derived based on the fan voltage and distance between the heater and sensor array. The R-squared values of five different fan voltages were calculated to be 99.17%, 99.71%, 99.23%, 99.50%, and 99.64% to demonstrate the accuracy of the derived formula. Accordingly, the measured and predicted maximum temperature curves were compared.

Finally, the amount of shift of the maximum temperature point on the +*z* axis owing to the effect of gravity was measured. The experimental results showed that as the fan voltage decreased and the distance between the heater and sensor array increased, the shift of the maximum temperature point towards the +*z* axis increased.

This study provides new insights into the heater manufacturing process. Implementation of the measurement system in the design, production, and testing stages of electric heaters will pave the way for the production of more efficient, economical, and homogeneous heaters. By increasing energy efficiency, the proposed system will contribute to a decrease in energy demand and, consequently, to the conservation of natural resources.

In the future, the number of sensors and surface area of the sensor grid can be increased to enhance the accuracy of the heating pattern. Additionally, positioning the sensors closer to each other improves the resolution of the obtained heating pattern. Furthermore, using the PT1000 for temperature measurement enhances the stability of the system and reduces power consumption. Finally, adding a low-noise amplifier to the temperature measurement circuit can improve the measurement accuracy and mitigate the disruptive effects of ambient noise on sensor voltage. The measurement plate was manually moved at 10 cm intervals, extending the measurement process and limiting the resolution in the *z*-axis. To eliminate these limitations, the measurement plate can be moved using a linear actuator or lead screw system in the future, allowing the plate position to be electronically controlled.

## Figures and Tables

**Table 1:** Sampling and communication commands.

S2	S1	S0	Command
1	1	1	Start sampling
0	0	1	Send sampled data: LCU1
0	1	0	Send sampled data: LCU2
0	1	1	Send sampled data: LCU3

**Table 2:** Three-dimensional heating patterns.

d_hs_ (cm)	Fan Voltage (V)				
	16	17	18	19	20
0	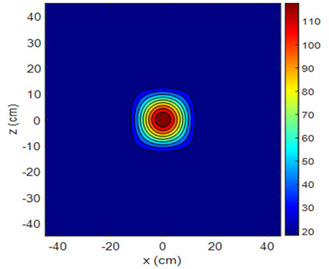	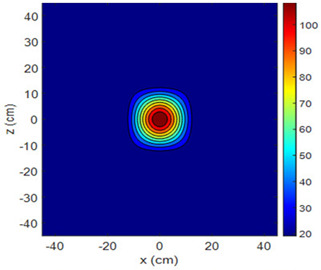	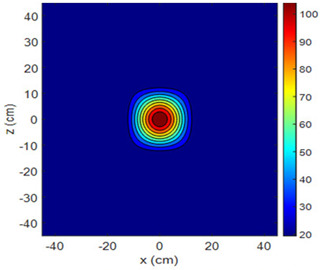	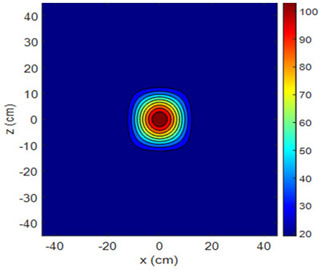	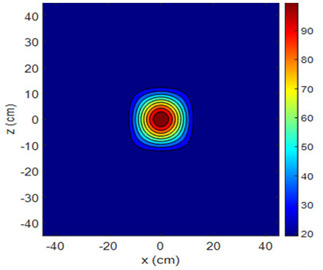
20	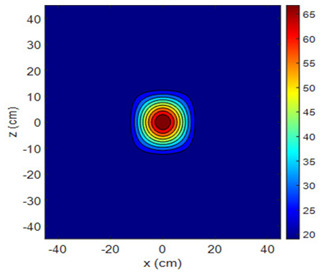	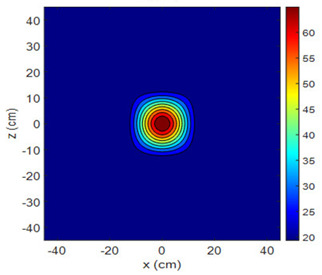	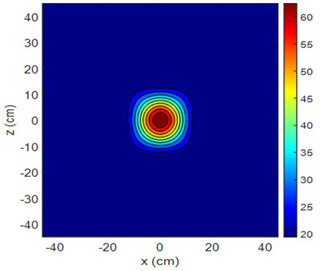	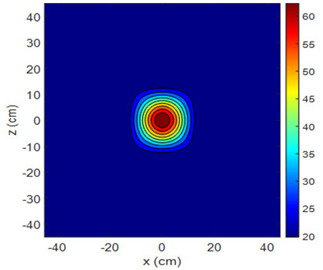	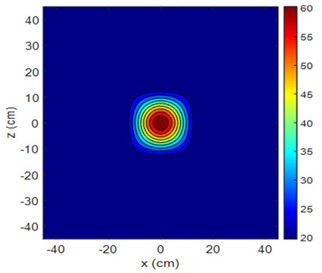
40	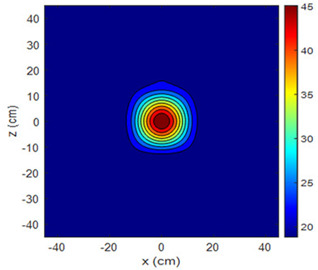	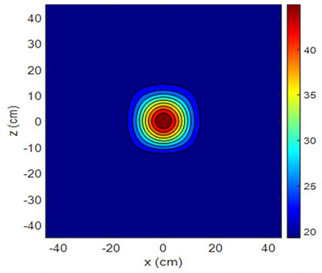	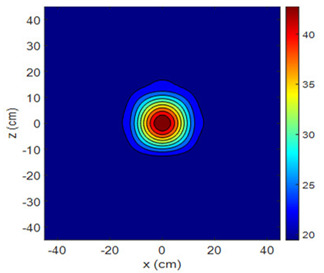	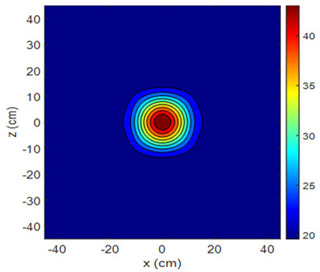	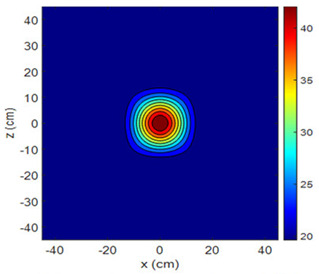
60	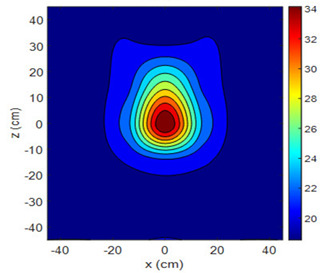	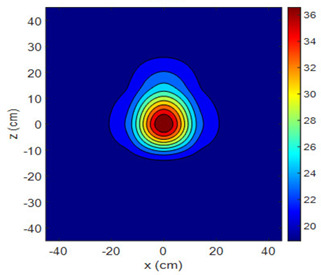	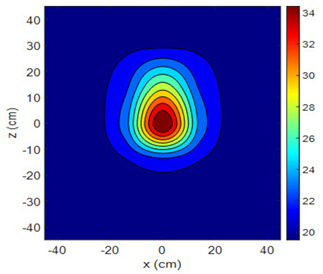	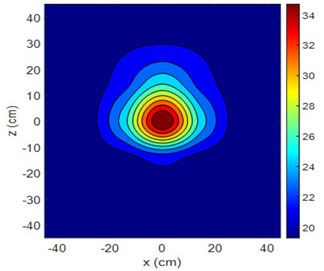	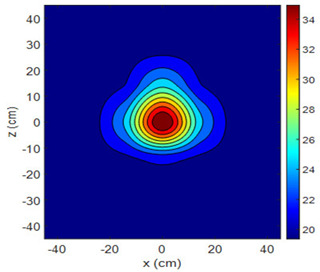
80	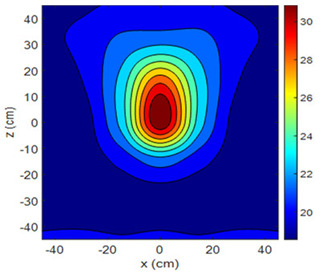	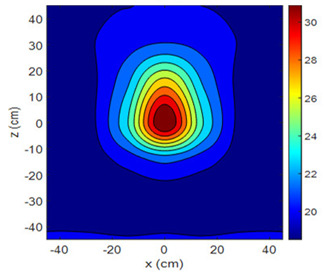	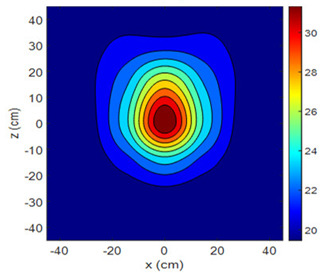	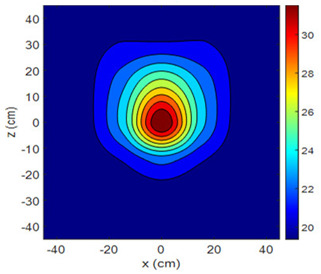	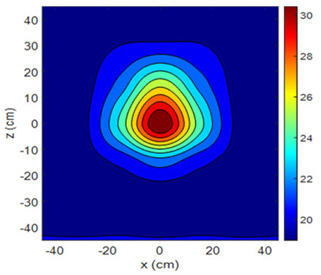
100	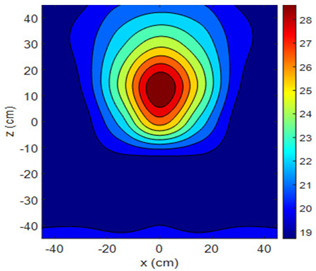	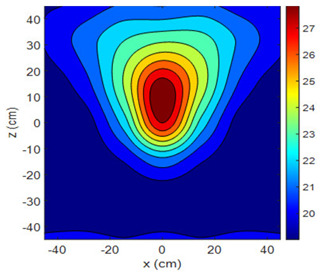	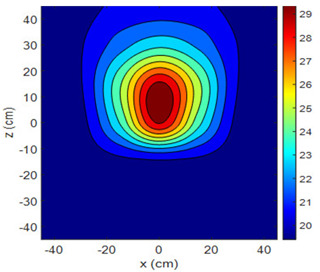	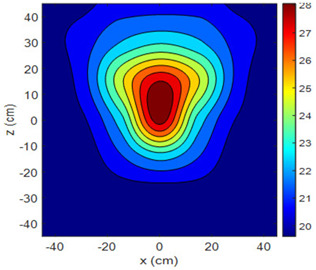	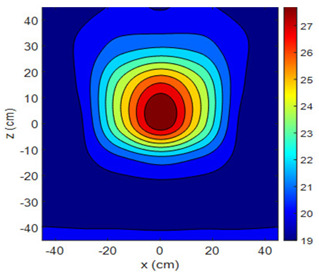

**Table 3:** Coefficients of maximum temperature equation.

Coeff.	Value	Coeff.	Value	Coeff.	Value
*c*_1_	173.8	*c*_4_	6.950 × 10^−2^	*c*_7_	−5.071 × 10^−4^
*c*_2_	−4.868	*c*_5_	8.146 × 10^−2^	*c*_8_	1.771 × 10^−6^
*c*_3_	−3.265	*c*_6_	−5.255 × 10^−4^	*c*_9_	−5.523 × 10^−8^

**Table 4:** Goodness-of-fit statistics.

Fan Voltage [V]	RMSE [°C]	RSQ
16	2.6935	0.9917
17	1.4414	0.9971
18	2.1745	0.9923
19	1.7418	0.9950
20	1.4149	0.9964

## Data Availability

The original contributions presented in the study are included in the article, further inquiries can be directed to the corresponding author.
